# Tracking your emotions: An eye-tracking study on reader’s engagement with perspective during text comprehension

**DOI:** 10.1177/1747021820905561

**Published:** 2020-02-27

**Authors:** Scarlett Child, Jane Oakhill, Alan Garnham

**Affiliations:** School of Psychology, University of Sussex, Brighton, UK

**Keywords:** Eye-tracking, pronouns, text comprehension, perspective

## Abstract

An eye-tracking study explored perspective effects on eye-movements during reading. We presented texts that included either a personal perspective (*you*) or an onlooker perspective (he or she). We measured whether fixations on the pronouns themselves differed as a function of perspective, and whether fixations on pronouns were affected by the emotional valence of the text which was either positive or negative. It was found that early in the text, processing of *you* is easier than *he* or *she*. However, as the character referred to by *he or she* becomes more familiar, fixations on *he or she* decrease, specifically in negative contexts.

## Introduction

When we read narrative texts, we monitor information about the protagonist (Creer et al., 2018; O’Brien & Albrecht, 1992; Zwaan, 1999). We use information about their goals and motivations (Zwaan, 1999) as well as information about their emotions (Child et al., 2018; de Vega et al., 1996; Gernsbacher et al., 1992; Gygax et al., 2003) to establish coherence and comprehend the text. Even though the protagonist is a central factor for narrative comprehension, there is a large body of evidence that readers do not automatically adopt the perspective of the protagonist (Creer et al., 2018; O’Brien & Albrecht, 1992). For example, O’Brien and Albrecht (1992) found that readers can use textual information that would not be available to the protagonist to arrive at a coherent text representation. Spatial violations from the perspective of the protagonist (someone coming *into* the health club when the protagonist is standing *outside* of the club, O’Brien & Albrecht, 1992) did not impact reading processes if readers were not specifically instructed to take the protagonist’s perspective. However, in conditions where readers were explicitly instructed to take the protagonist’s perspective, these spatial violations led to a disrupted reading process showing that prompts within the text can impact perspective taking during reading. For this study, we are interested in whether readers who *are* prompted to take the protagonist’s perspective do so stably throughout the text, or whether they revert to a more omniscient view during the course of reading.

Similar to studies by O’Brien and Albrecht (1992), studies by Creer et al. (2018) suggest that readers do not take the protagonist’s perspective unless prompted or instructed. They also provide evidence that readers who are instructed to, or manipulated into, taking the protagonist’s perspective are more sensitive to perspective relevant information and that this information is part of their mental representation of the text. Creer et al. (2018) also note that for readers to fully adopt the protagonist’s perspective, “it would require instances in which the reader would need to ignore their own knowledge, either from the text or general world knowledge.” As readers are unlikely to ignore or forget this contextual information (Creer et al., 2018), it is argued that perspective taking affects the way in which reader *validate* the information in the text against their mental representation but that it does not affect their attention during reading.

According to the RI-Val model (Cook & O’Brien, 2014; O’Brien & Cook, 2016), comprehension and the process of building a coherent text representation involve three processes: *resonance, integration*, and *validation*. In the resonance stage, existing information is reactivated from memory and this information is then integrated with the new (textual) information during the integration stage. In the validation stage, the linkages between previous and new information are then validated. Processing difficulty is determined by the fit between new and old information, that is, the better the fit, the easier the validation process (Cook et al., 2018). As suggested by Creer et al. (2018), readers are more sensitive to perspective relevant information when reading from the first-person perspective and they activate a wealth of detailed contextual information that has to be matched or validated against the information in the text. Due to the sensitivity to perspective relevant information, readers are able to detect even very subtle violations within the text. Hence, the detection of a mismatch between previous or contextual information (also world knowledge, Creer et al., 2018) and new information, disrupting the reading process, is more likely. We assume that the likelihood of detecting these anomalies during reading increases as readers proceed in the text, as more and more information has to be processed, integrated, and validated. Our study aims to explore whether readers who are prompted to adopt the protagonist’s perspective experience more disruptions as they progress through the text. If so, they might be less likely to upkeep a personal point of view during reading, to be better able to validate new information and arrive at a coherent text representation.

Literary theorists have proposed that readers do adopt the perspective of the protagonist through the use of the pronoun *you. You* is a *seduction to feel addressed* (Kacandes, 1991 in Schofield, 1998); however, the more information they process from this perspective, the more they realise that the *call is not quite accurate* (Kacandes, 1991 in Schofield, 1998). Readers might first be ready to adopt the protagonist’s perspective; however, as the validation process fails due to mismatches between their activated information, that is, their own previous world knowledge and the new information in the text, the reading process becomes more disrupted and the readiness to adopt the protagonist’s perspective fades. In our study, we aim to show that the effects of perspective on reading are not stable across the text, but that they change throughout the reading process. We predict that readers engage in perspective taking and take the perspective of the protagonist when texts are presented using the pronoun *you* at the beginning of the reading process. However, as they proceed to read the text, we assume that the mismatch between reactivated and new information causes readers to disengage from the personal perspective. As proposed by Creer et al. (2018), readers do not assume the perspective of protagonists presented from the third-person perspective. Hence, we assume that processing difficulty for the third-person perspective is more stable across the text than for the second-person perspective.

One way to manipulate the perspective within a text is to use different pronouns (e.g., I vs. you vs. he or she). For example, Creer et al. (2018) used the pronoun *I* to prompt readers to take the perspective of the protagonist, and the pronouns *he* or *she* to prompt an omniscient point of view. Even though some researchers have reported perspective effects, comparing texts including *I* or *he or she*, others have found conflicting evidence with regard to the first-person perspective (*I*) and its effect on the reader. A study by Brunyé et al. (2011) found that readers adopt the personal perspective (monitoring events from the perspective of the protagonist) through the use of the pronouns *I* and *you*. However, their results also show that the inclusion of additional information (more details about the character) causes readers to adopt the omniscient (external point of view, Brunyé et al., 2011) for texts including *I*. The study by Brunyé et al. (2011) shows that the degree to which readers engage with the perspective of the protagonist is not necessarily stable throughout the reading process. Also, the findings give evidence that some pronouns such as *you* are better prompts for readers to adopt the protagonist’s perspective than others (*I*), leading to a more stable and longitudinal engagement with the protagonist’s perspective. However, we assume that even when texts are presented from the *you* perspective, readers might struggle to maintain the perspective of the protagonist and, hence, shift to a more omniscient perspective during the course of reading as more and more information has to be validated and checked against their personal representation of the situation. To test this assumption, we will present texts written in the second-person *you* and the third-person *he or she* perspective and we will measure fixations at different points in the text to assess the readers’ abilities to integrate and validate new information.

The effects of the personal perspective, using the pronoun *you*, on reading and text engagement were investigated in a study by Child et al. (2018). The experiments presented in their paper give evidence that the personal perspective affects reading times as well as the content of readers’ mental representations. First, readers were found to process information faster when reading from a personal perspective (using the pronoun *you*); however, this effect was particular to positively valenced texts. The authors argued that readers engage in perspective taking, and monitor information from a personal perspective as long as this information is of a positive nature. In contrast, readers are reluctant to take the personal perspective and imagine a situation form their own viewpoint when they are faced with negative information. In a second study, an (in-)consistency paradigm was included so that a final explicit emotion at the end of the text was either consistent or inconsistent with the implicit emotion described in the text. Child et al. (2018) showed that reading latencies were similar for both perspectives as long as the explicit information matched the context, but that emotional inconsistencies caused increased processing difficulties for the personal perspective. The struggle to integrate mismatching information was particularly evident for the personal perspective and for texts describing negative situations, followed by an inconsistent positive outcome. Child et al. (2018) refer to research providing evidence that negative events trigger more empathic responses in individuals (Altmann et al., 2014; Keen, 2006; Kidd & Castano, 2013) and they suggest that this empathic engagement is enhanced through the use of the personal perspective, resulting in stronger mental representations of the text. The study by Child et al. (2018) underlines the role of perspective for narrative processing and also identifies the emotional valence as a factor influencing a reader’s readiness to engage with a text, their empathic engagement, as well as their mental representation.

The results of Child et al. (2018) demonstrate that there is an initial readiness to adopt the personal perspective for positive texts. However, perspective effects at the end of negative texts (for inconsistencies) suggest that the engagement with perspective (i.e., the empathic engagement with the character) might not be stable but changeable throughout the text. As previously indicated, readers might adopt a personal point of view initially, but as more information is processed, more personal knowledge is activated, and the validation process (see RI-Val model, Cook & O’Brien, 2014; O’Brien & Cook, 2016) is more likely to fail due to inconsistencies between information in text and a readers’ personal experiences. On the basis of the findings presented by Child et al. (2018), it might be assumed that perspective effects arise early in the text with readers adopting the personal perspective as soon as a positive emotion unfolds, but that they might be reluctant to take the personal viewpoint when negative information is presented. Child et al. (2018) reported reading time differences on the basis of average reading times per sentence within a paragraph; hence, their results do not give insights into how and when perspective effects arise.

Research so far has not given an insight into how long a reader’s reluctancy to take the protagonist’s perspective prevents a personal engagement and exactly how the empathic engagement with characters (either from a personal or onlooker perspective) changes perspective effects in the process of reading. For example, the lack of perspective effects for negative texts and faster reading times for the personal perspective in positive texts might not only be a result of an initial reluctance to engage personally with the text event, but of a change in the reader and their sensitivity to new information. However, the findings of our present study might indicate that for negative texts, including the personal perspective, there is an increased sensitivity to inconsistencies between experience and text information so that validation processes begin to fail early on and slow the reading process (Creer et al., 2018). This hypothesis is in line with the results reported by Creer et al. (2018, highlighting that attention to perspective relevant information leads to a sensitivity to violations between experiences and text information), but also with theories showing that negative emotions in the reader (assuming that readers usually mirror the protagonist’s emotions) lead to a greater scrutiny of the consistency of new information (Bless, 2000; Erber & Erber, 2001). We suggest that perspective engagement is affected by emotional valence and as emotions unfold throughout the text, and as new information has to be validated against a reader’s situation model or representation, perspective effects change throughout the reading experience.

Eye-tracking measurements have emerged as a useful tool to examine and unmask comprehension processes (Rayner, 2009). Fixation times are taken as an indicator of the actual time needed to process particular text elements (Carpenter & Just, 1977). First-pass measures of reading, for example, gaze duration, are associated with early processing stages (lexical access and encoding) and can vary as a function of lexical complexity or frequency. Also, the occurrence of unexpected text elements can lead to longer fixation durations due to encoding problems (Rayner, 1998). The pronoun *you* is not commonly used in narratives, and it might not be expected in a narrative context. Considering these frequency effects, longer fixations on the pronoun *you* might be expected. However, considering the findings of Child et al. (2018) with shorter reading times for texts including *you*, these frequency effects might be mitigated as readers readily adopt the personal perspective and are quickly drawn into the text (in particular for texts with a positive valence). Yet again, the time of course of these frequency and perspective effects has not been addressed by researchers thus far.

For our study, we are interested in not only whether early processing components are affected by perspective but also whether integration processes are facilitated through the use of the personal pronoun *you*. As argued by Rayner (1998), regressive eye-movements, that is, movements backwards to previous sections in the text (from right to left), can be associated with the reader’s attempt to link new information with previous information. Therefore, when readers detect inconsistencies between or have difficulties connecting earlier and more recent information, they engage in more backtracking, which leads to slower integration processes. We assume that perspective effects are not consistent across the text. In line with the findings by Creer et al. (2018) and considering the RI-Val model (O’Brien & Cook, 2016), we suggest that the reader’s engagement with the protagonist’s perspective is challenged by new information and by even small violations between new and previous information, including information that is based on readers’ knowledge and experiences. Hence, we suggest that the integration of new information gets more and more difficult throughout the text especially as readers attend to perspective relevant information as prompted by the use of the personal perspective. This tendency might be reflected by more frequent and longer regressions with the personal perspective at later stages in the text.

This study employs eye-tracking measures to gain insight into how perspective effects change through the text, and also to test whether these effects manifest early and/or late during processing. The study uses similarly emotional texts to those presented by Child et al. (2018) to prompt a more engaging reading process. Similar to Child et al. (2018), we will also administer emotional self-ratings to assess affective responses to text. We expect findings to be similar to those of Child et al. (2018), with more positive ratings for positive texts in the personal perspective. This finding would suggest that readers adopt the perspective of the protagonist at some point during reading (if not stably) and that they mirror their emotions and make this emotional experience a part of their overall text representation. Also, the lack of a perspective effect on emotional self-ratings for negative texts would suggest that the process of adopting the protagonist’s perspective (and emotion simulation) is disrupted in negative contexts, possibly due to a reluctance to engage with negative events from a personal point of view (Child et al., 2018).

## Method

### Participants

For this study, 44 undergraduate students of the University of Sussex were recruited (the data of two had to be excluded due to technical problems). Participants’ age ranged from 18 to 28 years with *M* = 20.39 and *SD* = 2.32. Before signing up electronically on the Sona recruitment platform of the university, and again before the experiment, participants were asked about their first language and their reading ability. Individuals who were not English native speakers or who showed indications of reading problems or disabilities were excluded from the experiment. Participants received course credit or money for their participation.

Our sample size was justified by an a priori power analysis performed in G*power (Faul et al., 2007). It was calculated that for α = .05 and 1 – β = .95 and an estimated medium effect size (*f* = 0.25; Cohen, 1969), a sample size of 36 would be needed.

### Apparatus

Eye-movements were recorded via a table-mounted infrared camera and an SR-Eyelink 1000 eye-tracker (SR Research, Ottawa, Canada) with a sampling rate of 1,000 Hz. Eye-movements were recorded from the right eye of each individual. Items were presented using the Experiment Builder software (SR Research) on a 21.5 in. monitor (iMac, with Windows XP 2002 operating system). Participants were asked to sit so that they could place their head in the chinrest and forehead restraint which was placed at about 60 cm from the screen to minimise head movements. Before the start of the experiment, a 13-point spherical calibration was performed (to a 0.5° calibration average). A drift check was carried out before the start of each new item, and recalibration was performed, if necessary.

### Items

Twenty-four experimental items were taken from the study by Child et al. (2018) and eight similar items were generated to arrive at a total of 32 items. The lengths of the passages ranged from 47 to 96 words (251–501 characters), with *M* = 70.94, *SD* = 13.05 (for characters: *M* = 375.11, *SD* = 68.49). Texts presented a character experiencing either a negative or a positive situation (i.e., 16 items of 32 in each valence). Throughout the text, the emotion unfolded only implicitly (see Example 1).

Example 1.

1a. “*You*” perspective: *With a full bag in your hand, you make your way home. It feels quite heavy, but that does not really matter. You had assumed that you would have to spend so much more today. You had been trying to save up for a while, and this was a real bargain. You look at your bag with great satisfaction.*

1b. “*He/She*” perspective: *With a full bag in his hand, Peter makes his way home. It feels quite heavy, but that does not really matter. He had assumed that he would have to spend so much more today. He had been trying to save up for a while, and this was a real bargain. He looks at his bag with great satisfaction.*

The final sentence always contained an explicit emotion word reflecting the valence of the text. Each text occurred in both perspective conditions, including either the personal pronoun *you or*, for the onlooker perspective, containing a proper name for the first mention of the character followed by the pronouns *he* or *she. For items including the onlooker perspective, the gender of the characters was counterbalanced across items*.

Texts were separated into multiple interest areas (IAs), each of which included a pronoun (i.e., either you or he or she). An individual IA also included words adjacent to the actual pronoun. For IAs (pronouns) that were in the middle of a sentence and had adjacent words that were part of the same sentence, we included plus or minus one word in the IA. For pronouns starting a sentence, only the following word was included, for sentences ending with a pronoun, only the preceding word was included, so that regions would not cross sentence boundaries. We included these words in the IAs because previous researchers have shown that readers are likely to skip pronouns (function words) during reading (e.g., Rayner, 1998).^1^ We assigned ordinal numbers to each pronoun area in the text (e.g., the first pronoun was included as 1, see Example 1, underlined areas, seven IAs in total in this example). Texts included up to 14 pronouns (*M* = 7.22, *SD* = 2.47). Pronoun areas (position, word number, and length) did not differ between perspective conditions. Twenty-four items were added as distractors. Distractor (filler) items were taken from Gygax et al.’s (2003) study and rewritten so that half included the third-person perspective and the other half included the first-person “I” perspective. Fillers also referred to emotional situations but were ambiguous in their outcome.

### Design

The study followed a 2 × 2 mixed-measures design, with valence (a within factor for participants, and a between factor for items; negative vs. positive) and perspective (*you* vs. *he or she*, within for both participants and items) as factors (Child et al., 2018). We also included the IA as a continuous factor (ordinal, 14 levels, ranging from 1 to 14).^2^ Each participant was presented with one of two lists (each item in one of two versions, with the pronoun *you* are *he or she*), following a Latin Square Design, with each list containing the same number of items (32 experimental items plus 24 filler items) and the same number of experimental items in each condition (eight items per condition). Each item only occurred once per list, including either the pronoun you or he or she. The number (and length) of pronoun areas did not differ between lists.

### Procedure

Items were individually presented in a different randomised order for each participant. Participants were asked to read the texts and press a button on the keyboard after reading. After this response, participants were asked to rate their own emotional response to the text on a scale from 1 (*negative*) to 10 (*positive*). Ratings were given on a rating bar (on a continuous line) using the mouse. To proceed to the next item, participants were asked to click on a proceed button again using the mouse. The next trial started as soon as participants focused on the black dot appearing for the drift check.

## Results

### Eye-movement data

The data were extracted using the fixation report function in the Data Viewer software and eye-tracking measures were obtained through the Get Reading Measures script provided by SR Research (2011). It is suggested that readers are not able to fully process text in less than 50 ms (Inhoff & Radach, 1998; Jegerski & VanPatten, 2013). Therefore, we excluded fixations of less than 50 ms from the further analysis. Linear mixed effect models were used to analyse the remaining data. The analysis was run in R (R Core Team, 2013, version 3.4.3.) using the lme4 package (Bates et al., 2014) and lmeTest (Kuznetsova et al., 2015) for Satterthwaite approximations for the degrees of freedom. Perspective (personal *you*; onlooker *he or she*), valence (*negative/positive*), and finally the pronoun area were included as fixed factors. Participants as well as items were included as random factors with both intercepts and slopes included where possible.^3^ The default restricted maximum likelihood estimations (provided by the lme4 package) were used. To assess model fit (using maximum likelihood estimations), models that satisfied the convergence criteria were compared using the Akaike information criterion (AIC; Bates, 2010). We also carried out a principal component analysis for each of the sets of random effects to check for overparametisation (RePsychLing package, Baayen et al., 2015). We report models with the lowest calculated AICs and that were not subject to overparametisation (Bates et al., 2018). We used the sjPlot package to crate tables for linear mixed models (LMM) coefficients (Lüdecke, 2018). Contrasts were set using sum contrasts.

Effects of perspective and of valence might affect the processing of new information at different stages such as encoding only or integration only, or effects might be evident for processing more generally. Hence, we analysed three eye-tracking measures (Liversedge et al., 1998): gaze duration (the sum of all fixation in a region until that region is exited to the left or right) to reflect encoding processes, regression-path duration (all fixations until a region is exited to the right) to reflect integration processes, and the total duration (i.e., sum of all fixations on a region). We found main effects of all three factors on gaze durations (see Table 1).

**Table 1.** LMM coefficients and effects of perspective (you/he or she), valence (negative/positive), and pronoun region on gaze duration.

	Gaze duration		
	*B*	CI	*p*
Fixed parts			
(Intercept)	342.95	[322.63, 363.26]	<.001
Valence	−15.43	[−29.40, −1.47]	.035
Perspective	−35.52	[−46.30, −24.75]	<.001
Pronoun region	−4.51	[−6.44, −2.58]	<.001
Valence:Perspective	15.12	[4.35, 25.89]	.007
Valence:Pronoun Region	3.64	[1.71, 5.57]	<.001
Perspective:Pronoun Region	6.61	[4.71, 8.50]	<.001
Valence:Perspective:Pronoun Region	−3.77	[−5.65, −1.89]	<.001
Random parts			
σ^2^	32,783.985		
τ_00, part_	2,364.695		
τ_00, trial_	969.207		
ρ_01_			
*N*_part_	42		
*N*_trial_	32		
ICC_part_	.065		
ICC_trial_	.027		
Observations	6,220		
*R*^2^/ $\Omega_{0}^{2}$	.119/.118		

LMM: linear mixed models; CI: confidence interval; ICC: Intra-Class-Correlation-Coefficient.

**Figure 1.**
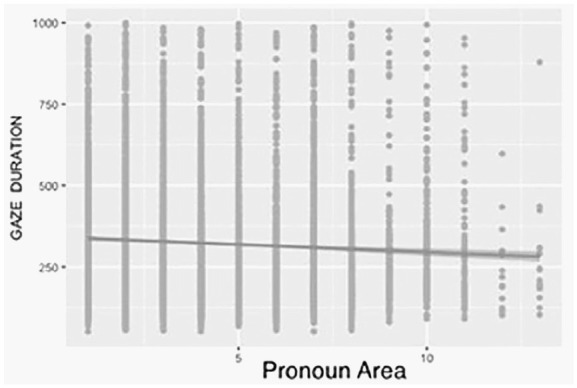
Gaze durations for individual pronoun areas (1–14).

For perspective, the occurrence of the pronoun *you* lead to faster processing compared with the pronouns *he or she*. The effect of perspective in the fitted model is 2 × *B* (see Table 1) for perspective, which is 71.04 ms. For the valence of the text, positive texts had shorter gaze durations than negative texts (Δ = 30.86 ms). For the pronoun areas, gaze duration decreased with the ordinal position of the pronouns, that is, later occurrences of the pronoun were fixated on for shorter periods (see Figure 1).

**Figure 2.**
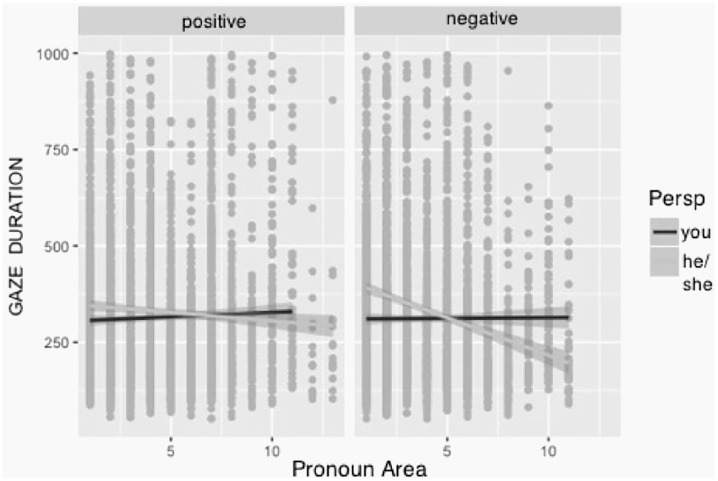
Gaze durations for pronoun areas by valence and perspective.

We found a three-way interaction of all factors (valence, pronoun region, and perspective, see Table 1). As can be seen in Figure 2, the interaction between pronoun region and valence was prominent in the onlooker perspective; however, the personal perspective fixations remained similar for negative and positive texts across regions.

The results so far indicate that the perspective, valence, and the reoccurrence of pronouns influence reading behaviour during early processing. A similar pattern of results was found for the regression-path duration (all fixations on a region and regressive regions until the region is exited in a progressive manner, see Table 2).

**Table 2.** LMM coefficients and effects of perspective (you/he or she), valence (negative/positive), and pronoun region on regression-path duration.

	Regression-path duration		
	*B*	CI	*p*
Fixed parts			
(Intercept)	376.23	[352.38, 400.08]	<.001
Valence	−0.50	[−19.02, 18.02]	.958
Perspective	−26.84	[−40.23, −13.44]	<.001
Pronoun region	−1.72	[−3.92, 0.47]	.124
Valence:Perspective	13.67	[0.29, 27.06]	.050
Valence:Pronoun Region	−0.29	[−2.48, 1.90]	.797
Perspective:Pronoun Region	4.32	[2.16, 6.48]	<.001
Valence:Perspective:Pronoun Region	−2.55	[−4.70, −0.40]	.020
Random parts			
σ^2^	38,050.842		
τ_00, part_	2,448.343		
τ_00, trial_	2,028.107		
ρ_01_			
*N*_part_	42		
*N*_trial_	32		
ICC_part_	.058		
ICC_trial_	.048		
Observations	5,763		
*R*^2^/ $\Omega_{0}^{2}$	.119/.118		

LMM: linear mixed models; CI: confidence interval; ICC: Intra-Class-Correlation-Coefficient.

**Figure 3.**
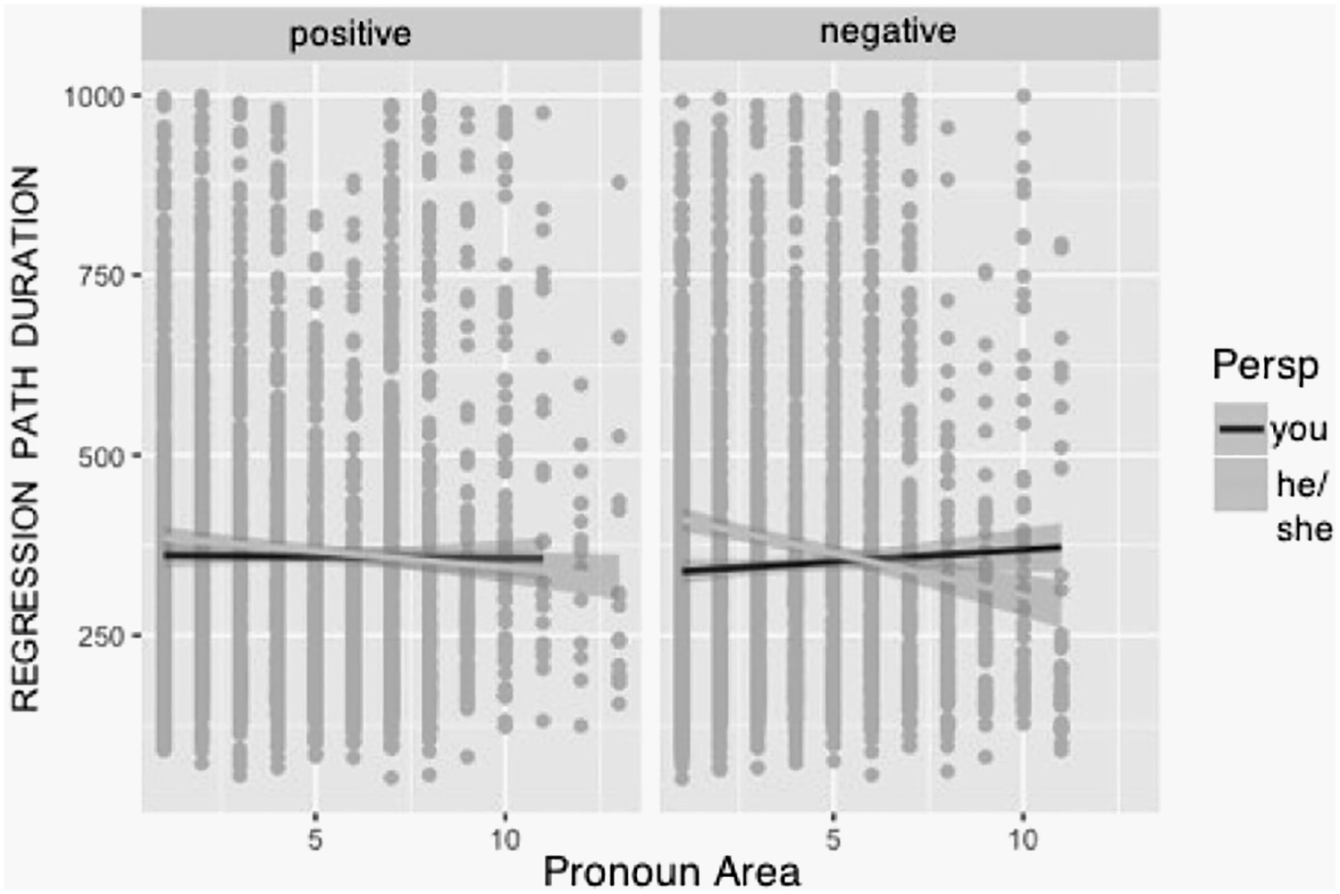
Regression-path duration for pronoun areas by valence and perspective.

Again, we found the perspective effect with shorter times for the personal perspective (Δ = 53.68). For the regression-path duration, we did not find main effects of valence or pronoun area, and the interaction of those two variables was not significant for this measure. Again, we found a three-way interaction between all measures (see Table 2) which is shown in Figure 3.

**Figure 4.**
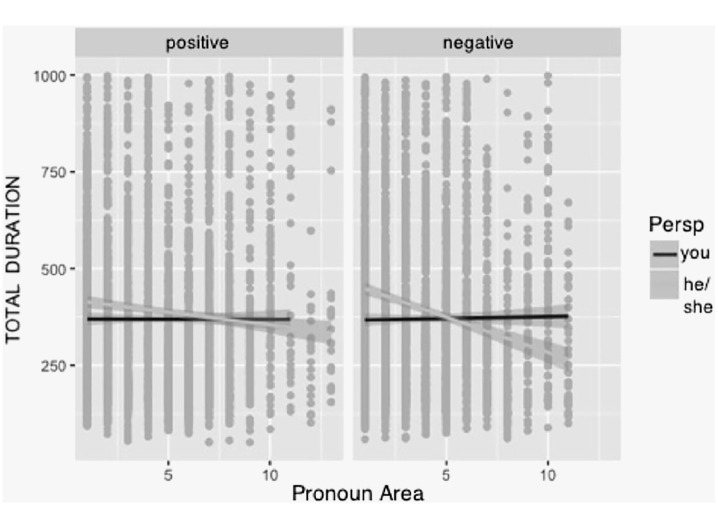
Total durations for pronoun areas by valence and perspective.

Finally, we analysed the total duration (see Table 3; summing up all fixations in that region). Times were again shorter for the personal perspective as compared with the onlooker perspective (Δ = 73.42) and reading times decreased as the text proceeded. For the total fixation duration, we did not find a main effect of valence (see Table 3). Again, the three-way interaction of perspective, valence, and pronoun area was evident (see Table 3; Figure 4). For negative texts, participants read first faster for the *you* perspective, but as the text proceeds, reading times became faster for the onlooker perspective (*he or she*).

**Table 3.** LMM coefficients and effects of perspective (you/he or she), valence (negative/positive), and pronoun region on total duration.

	Total duration		
	*B*	CI	*p*
Fixed parts			
(Intercept)	407.46	[384.02, 430.91]	<.001
Valence	−10.64	[−27.06, 5.78]	.210
Perspective	−36.71	[−49.16, −24.25]	<.001
Pronoun region	−5.48	[−7.56, −3.39]	<.001
Valence:Perspective	11.34	[−1.11, 23.78]	.079
Valence:Pronoun Region	2.66	[0.57, 4.74]	.013
Perspective:Pronoun Region	6.30	[4.26, 8.35]	<.001
Valence:Perspective:Pronoun Region	−3.15	[−5.19, −1.11]	.002
Random parts			
σ^2^	39,938.853		
τ_00, part_	3,049.546		
τ_00, trial_	1,467.570		
ρ_01_			
*N*_part_	42		
*N*_trial_	32		
ICC_part_	0.069		
ICC_trial_	0.033		
Observations	6,492		
*R*^2^/ $\Omega_{0}^{2}$	.123/.122		

LMM: linear mixed models; CI: confidence interval; ICC: Intra-Class-Correlation-Coefficient.

For all measures, we found a perspective effect with shorter times for the *you* perspective compared with the onlooker perspective. For most measures (except for regression-path duration), we found an effect of pronoun area, with shorter times for the pronoun region as the text proceeded. For all measures, the three-way interaction between the three factors (perspective, valence, and pronoun area) was significant, showing that the interaction of valence and pronoun area was specific to the onlooker perspective. For the pronouns he or she, readers’ times decreased as the text proceeded. We found evidence that the pronouns that are used in text (prompting readers to either take a personal perspective or onlooker perspective) affect early and late processing stages. We also found that times to process pronouns that occur in negative and positive texts and times to process pronouns that occur at different times in the text differ as a function of perspective.

### Emotional responses

We used the same procedure and type of analysis as for the eye-movement data, except that pronoun region was not a factor in this analysis. Participants rated their emotions on a scale from 0 to 10 (how happy the text made them feel, 0 = *not happy at all*; 10 = *very happy*, integer scale). Individuals’ emotional response was in line with the texts’ valence; that is, they rated their own emotions more positive for positive texts and more negative after having read negative texts (Δ = 4.42 ms, see Table 4).

**Table 4.** LMM coefficients and effects of perspective (you/he or she), valence (negative/positive), and pronoun region on emotional responses.

	Emotional responses		
	*B*	CI	*P*
Fixed parts			
(Intercept)	4.7	[4.57, 4.92]	<.001
Valence	2.21	[2.00, 2.42]	<.001
Perspective	0.17	[−0.59, 0.92]	.670
Valence:Perspective	1.38	[0.63, 2.13]	<.001
Random parts			
σ^2^	19.4		
τ_00, part_	0.63		
τ_00, trial_	3.21		
ρ_01_	1.000		
*N*_part_	42		
*N*_trial_	32		
ICC_part_	0.027		
ICC_trial_	0.138		
Observations	1,327		
*R*^2^/ $\Omega_{0}^{2}$	.740/.740		

LMM: linear mixed models; CI: confidence interval; ICC: Intra-Class-Correlation-Coefficient.

**Figure 5.**
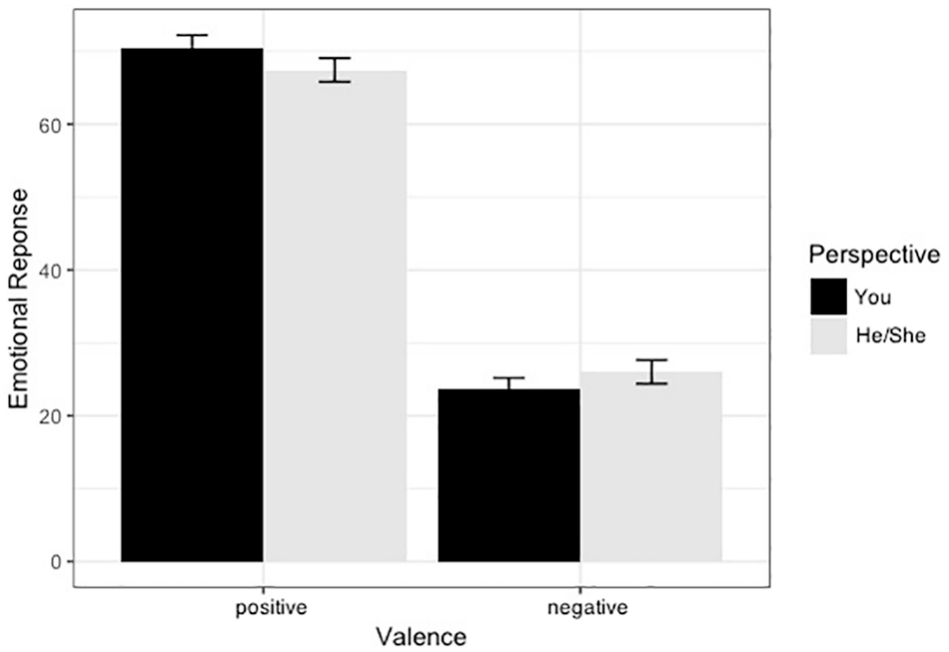
Emotional responses for valence (positive/negative) and perspectives (you and he or she, ±1 *SE*).

Also, for negative texts, participants rated their own emotions similarly for texts including *you* and texts including *he or she*. For positive texts, the personal perspective led to more positive emotional responses than the onlooker perspective, β = 3.09, *SE* = 1.09, *t*(871) = 2.84, *p* = .024. The interaction between valence and perspective was significant. For texts including the pronoun *you*, participants’ emotional ratings were overall more strongly in line with the valence of the text (see Figure 5).

## Discussion

Our study investigated reading behaviour as a function of readers’ engagement with a text, as determined by perspective. Our study is (to our knowledge) one of the first that gives evidence that perspective effects are evident directly at the pronoun and that these effects change throughout the reading process. In our eye-tracking experiment, IAs including personal pronouns were analysed and it was found that the perspective (the use of the pronoun *you* or *he or she*) affected early and late processing stages in the pronoun areas.

For early measures of processing (gaze durations), gaze durations decreased as readers progressed through the text. However, taking into account the perspective, this decrease was only evident for the omniscient perspective, that is, pronoun areas including *he or she*, whereas gaze durations were stable across the text for areas including *you*. For the first few pronoun areas of a text, readers fixated longer on *he or she* than on *you*; however, as the text proceeded, and durations for *he or she* decreased, gaze durations on late or final pronoun areas were shorter for *he or she* than for *you*. The finding is to some extent surprising as the pronoun *you* does not occur very commonly in narrative contexts (as opposed to *he or she*), and this lack of frequency should be reflected in longer gaze durations. However, shorter fixations on *you* are in line with our prediction that, initially, readers readily adopt the perspective of the protagonist, and that frequency effects are mitigated by a higher motivation to proceed in the text.

As opposed to pronoun areas including the pronouns *he or she*, gaze duration on *you* did not decrease throughout the text. The speed up with regard to the processing of *he* or *she* can be explained by an adjustment to the pronoun use within the text in case of the more common omniscient perspective; however, this explanation on its own cannot account for shorter gaze durations on *you* for the first few regions. Another explanation for the decrease in durations for *he or she* is that as the text goes on, and information about the protagonist unfolds, readers become more familiar with and adjust to the character so new information about them is anticipated and can be encoded more rapidly in line with the reader’s expectations, which is a process that is not necessary for the second-person perspective. This effect might also be supported by the general familiarity to the third-person pronouns in text. Readers’ expectations might be particularly strong for negative events as their level of empathy is suggested to be higher than for neutral or positive events (Altmann et al., 2014; Keen, 2006; Kidd & Castano, 2013).

In case of the personal perspective, we suggested that readers are initially receptive of perspective relevant information and have a sensitivity to information relating to *you*. Child et al. (2018) found evidence that readers processed information about *you* with greater ease due to a greater engagement with the characters’ emotions in text. Even though a greater familiarity to and expectations towards the pronouns *he* or *she* would suggest a greater initial ease in case of the third-person perspective, the occurrence of *you* led to faster processing encoding. Child et al.’s findings are in line with our results, however, only for the first pronoun regions of a text. Readers do not show the same ease of encoding for later pronoun regions as they do for the omniscient perspective. We suggest that the initial readiness to adopt the protagonist’s perspective or interest in perspective relevant information fades throughout the reading process as more and more information is validated. The validation of new information fails as conflicts between the text representation and the reader’s personal knowledge and previously established situation models become apparent (Cook & O’Brien, 2014; O’Brien & Cook, 2016). Readers might revert to a more omniscient perspective, which makes the occurrence of the pronoun *you* difficult to accommodate and encode, and hence, processing is not facilitated.

Another explanation might be that due to the unfamiliar encounter of the pronoun *you* in the text, readers engage in less effortful processing initially which would result in poorer text comprehension. As the text proceeds, readers ignore or get accustomed to the uncommon pronoun use, but their adjustment to *you* is still more difficult than the adjustment to the omniscient perspective. This would mean that frequency and familiarity effects occur later in the text and affect processing ease. Taking into account the rating data in which readers had to rate their own emotions at the end of the text, ratings were more in line with the emotion in the text for texts including you, which would speak against a speed-accuracy trade-off and poorer comprehension due to text skimming.

The pattern of results found for early processing measures was similar to those found for late measures, that is, regression-path duration and total duration. We suggested that readers adjust to characters referred to by *he or she* and that they track their emotions. New information is encoded with increasing ease, as it can be linked to previous information given by the context of the story (see RI-Val model). We only presented paragraphs that presented emotional information that was in line with the context (as opposed to Child et al., 2018; Gygax et al., 2003) so readers were able to use contextual information to integrate new information fairly easily—with more of this contextual information helping the integration process at later stages in the text.

Another explanation for the greater ease of integration at later stages in the text for *he* and *she* is that readers are first challenged with resolving anaphoric references and linking these pronouns to the protagonist (a process which is not necessary for *you*). However, this explanation does not account for valence effects during the integration process. The data presented in this experiment lead us to suggest that the empathic relationship between the character and the reader play a role in integration, and that this relationship is stronger when characters experience negative emotions (Altmann et al., 2014; Keen, 2006; Kidd & Castano, 2013). Emotional information can be used to link different parts of a text (de Vega et al., 1996) and it can help readers understand a character’s actions and goals (Zwaan, 1999). The data in this experiment suggests that readers are particularly good at linking or tracking negatively valenced emotional information and that this information can help the integration process especially at later stages in the text once the emotional valence unfolds.

The integration of the pronoun *you* did not get easier as readers progressed through the text. Again, initially the integration of you was faster than the integration of *he* or *she*. As frequency effects mainly affect early processing stages (lexical processing, for example, Inhoff & Rayner, 1986), we suggest that results are due to a greater sensitivity to perspective relevant information for the second-person perspective (Creer et al., 2018). Readers then activate a wealth of information in connection to the text, their own experiences, and knowledge. Their sensitivity to violations between new information and old information challenges the validation process (Creer et al., 2018), and hence the pronoun becomes more difficult to integrate. Also, the reader’s empathic engagement with the character might be more difficult as some of the protagonist’s emotional responses or their actions might not correspond to readers’ personal experiences or expectations. Therefore, the processing or integration of information connected to *you* does not fall into the same ease as for texts including the omniscient perspective.

Eye-tracking measures suggest that initially readers do take the personal perspective of the protagonist when prompted through the use of the pronoun *you*, but that as they read on in the text, perspective taking might hinder different stages of processing due to failing validation processes. Similar to Child et al. (2018), we also analysed whether emotional responses, provided by readers after text processing, are affected by perspective. Our results were similar to the ones reported by Child et al. (2018). Ratings were more in line with the emotion expressed in the text for the personal perspective and, in particular, ratings were higher for texts including *you* when the situation in the text was of a positive nature. We suggest that readers mirror positive emotions in texts when prompted to assume the protagonist’s perspective, but that they are reluctant to mirror negative emotions.

The difference between our eye-tracking measures and the self-ratings is interesting as findings suggest that the processes of building up empathy for a(-nother) character (*he or she*, that is, cognitive empathy) and of mirroring an emotion through adopting the character’s perspective (i.e., affective empathy) are to some extent independent, which is in line with studies suggesting a double dissociation between cognitive and affective theory of mind (e.g., Kalbe et al., 2010). Readers adopted (at least positive) emotions from texts including *you*; however, there was no evidence that their empathic engagement with the character (*you*) affected processing ease (as opposed to the omniscient perspective). This suggestion could be pursued in future studies, which might investigate whether readers’ affective responses to text (through the use of perspective) impact on the way they activate and use personal knowledge or experiences during processing. Following the notion by Creer et al. (2018), a readers’ sensitivity to perspective relevant information might lead to validation problems. Readers’ problems in validating new information and their own affective responses (that might be different to the emotions of character in text) could then lead to difficulties in building an empathic relationship with the character.

Our study investigated eye-movements as a function of perspective and valence and found that the engagement with perspective in text is not only subject to the emotions experienced by the characters but also by how far readers had progressed within a text. Further research should investigate the interactions between reader’s own emotions and experiences with those of a protagonist and explore whether common characteristics can lead to more stable perspective taking effects.

## Notes

1. We also set an interest area for the emotion word at the end of the passage to examine effects of perspective on that region. However, we did not find perspective (or valence) effects for that area.

2. Due to the different numbers of pronoun areas across items, we tested whether reading measures were affected by whether texts were long or short, that is, whether the number of pronoun areas was below or above the median number of areas of all texts (with *Mdn* = 7, texts with seven areas were omitted for this analysis). We did not find an effect of text length or interactions between text length (short or long) and valence or perspective.

3. Some models did not converge when random slopes were included. Where this was the case, we report more restricted models including combinations of random intercepts and slopes that were not affected by convergence issues (Angele et al., 2014).
